# Astigmatic correction of simultaneous femtosecond laser-assisted cataract surgery (FLACS) with intrastromal arcuate keratotomy (ISAK) versus Toric intraocular Lens Impantation with conventional phacoemulsification

**DOI:** 10.1186/s12886-021-02059-2

**Published:** 2021-08-14

**Authors:** Hye Ji Kwon, Hun Lee, Jin Ah. Lee, Jae Yong Kim, Hungwon Tchah

**Affiliations:** Department of Ophthalmology, Asan Medical Center, University of Ulsan College of Medicine, 88, Olympic-Ro 43-Gil, Songpa-Gu, Seoul, 05505 South Korea

**Keywords:** Femtosecond laser-assisted cataract surgery, Femtosecond laser-assisted intrastromal arcuate keratotomy, Toric intraocular lens implantation, Astigmatism, Cataract surgery

## Abstract

**Background:**

To compare the efficacies in astigmatic correction of simultaneous femtosecond laser-assisted cataract surgery (FLACS) with intrastromal arcuate keratotomy (ISAK) versus toric intraocular lens (IOL) implantation with conventional phacoemulsification in moderate astigmatism.

**Methods:**

A retrospective chart review was conducted for patients who had undergone cataract surgery by one surgeon. We identified patients with preoperative corneal astigmatism from + 0.75 to + 2.00 diopters (D) who had undergone astigmatic correction with FLACS with ISAK or toric IOL implantation with conventional phacoemulsification. We measured the visual acuity, intraocular pressure, automated keratometer, manifest refraction, and topography preoperatively and 1-day, 1-month, 3-month, and 6-month postoperatively. The vector analysis of refractive astigmatism was performed.

**Results:**

Of a total of 48 eyes of 48 patients, 27 eyes of 27 patients had FLACS with ISAK (AK group), and 21 eyes of 21 patients had conventional cataract surgery with toric IOL implantation (toric IOL group). Refractive astigmatism was significantly decreased in both groups. The mean preoperative and 6-month postoperative refractive astigmatism were 1.85 ± 1.07 and 0.99 ± 0.51 D, respectively, in the AK group (*P* = 0.028), and 1.84 ± 0.81 and 0.68 ± 0.21 D, respectively, in the toric IOL group (*P* < 0.001). There was no significant difference in refractive astigmatism between the two groups at 6-month postoperatively (0.99 ± 0.51 vs 0.68 ± 0.21 D, *P* = 0.057). At 6-month postoperatively, parameters for vector analysis of refractive astigmatism showed no statistical difference between the two groups. Corneal astigmatism was significantly decreased in the AK group. Corneal astigmatism from topography and the automated keratometer were significantly lower in the AK group 6-month postoperatively compared to toric IOL group (0.94 ± 0.40 vs. 1.53 ± 0.46 D, *P* = 0.018 for topography; and 0.98 ± 0.69 vs. 1.37 ± 0.41 D, *P* = 0.032 for the automated keratometer).

**Conclusions:**

FLACS with ISAK could be an effective procedure for reducing astigmatism as well as toric IOL implantation in cataract surgery.

## Introduction

A significant number of patients undergoing cataract surgery tend to have a varying degree of corneal astigmatism [1, 2]. Approximately, a third of cataract patients have more than 1.25 diopters (D) of preexisting corneal astigmatism, whereas approximately 10% have 2.00 D or higher [1–6]. The reduction of refractive astigmatism after cataract surgery can result in a significant improvement in visual quality, but residual astigmatism decrease visual acuity and quality of vision [7, 8]. Postoperative astigmatism is an important cause for the lack of achieving emmetropia even after routine cataract surgery [9, 10].

Over the past decade, various approaches, including limbal relaxing incisions, corneal incision on the steep axis, astigmatic refractive keratectomy, and toric intraocular lens (IOL) implantation, have been employed to reduce preexisting astigmatism during cataract surgery [10–12]. However, limbal relaxing incisions have limitations that include restricted predictability, induced irregular astigmatism, induced ocular aberrations, and abnormal wound healing [11, 12]. Excimer laser procedures after cataract surgery are useful for eliminating residual astigmatism but are rarely used because of availability and ocular surface problems following excimer laser treatment [13]. Toric IOL implantation during cataract surgery allows better management of astigmatism, leading to better uncorrected visual acuity [14, 15].

Femtosecond laser-assisted intrastromal arcuate keratotomy (ISAK), demonstrating higher precision, safety, and reproducibility in reducing refractive astigmatism, has been suggested as an alternative to these surgical techniques [16, 17]. Along with the introduction of femtosecond laser-assisted cataract surgery (FLACS), cataract surgeons could perform femtosecond laser-assisted ISAK during FLACS to reduce corneal astigmatism [18–22]. Our previous study observed no difference in postoperative astigmatism correction between femtosecond laser-assisted trans-epithelial arcuate keratotomy (AK) after cataract surgery for residual astigmatism and conventional cataract surgery with toric IOL implantation [19]. The aim of the present study was to compare the efficacies in astigmatic correction of simultaneous FLACS with ISAK versus toric IOL implantation with conventional phacoemulsification in patients with moderate astigmatism.

## Methods

We conducted this retrospective study with the approval of the Institutional Review Board of the Asan Medical Center and the University of Ulsan College of Medicine, Seoul, South Korea (2019–1244). The study adhered to the tenets of the Declaration of Helsinki and followed good clinical practice. All patients were informed of risks and benefits of the surgery and provided written consent for surgery.

A retrospective chart review was conducted for patients who had undergone cataract surgery by one surgeon (HT) at the cataract and refractive clinic of the Asan Medical Center. We identified those who had undergone astigmatic correction through FLACS with ISAK or toric IOL implantation with conventional phacoemulsification. Medical records of those who had completed all follow-up visits for 6 months (1-, 3-, and 6-month postoperatively) were reviewed. Patients who met the following inclusion criteria were included: age above 18 years, the presence of preexisting corneal astigmatism ranging from + 0.75 to + 2.00 D, and agreement of FLACS with ISAK or toric IOL implantation during cataract surgery. Only one eye per patient was included. For patients who had undergone cataract surgeries in both eyes, the first eye was included. Patients were excluded from the analyses if they had any optical opacities or pathologies on slit-lamp examination, previous corneal surgeries, ocular trauma, intraocular surgery, severe dry eye, corneal disease, ocular infection, or collagen vascular/autoimmune diseases. Among all the finally included patients, those who had undergone FLACS with ISAK were categorized under the AK group and those who had undergone cataract surgery with implantation of toric IOL were categorized under the toric IOL group.

All surgeries were performed by one surgeon using topical anesthesia (0.5% proparacaine hydrochloride). Cataract surgeries were performed with phacoemulsification and implantation of a foldable IOL with 2.2 mm limbal incision. The incisions were located at the steep axis of cornea measured by automated keratometer. All non-toric or toric IOLs were injected into the capsular bag. Main incisions were sealed with anterior stromal hydration without corneal sutures. Topical antibiotics and steroid eyedrops were used for 1-month after surgery.

In the AK group, all patients underwent cataract surgery using the Catalys femtosecond laser platform (Abbott Medical Optics, Inc., Santa Ana CA, USA). Horizontal limbal marker was done in sitting position prior to the laser to avoid cyclotorsion. After the patient lies supine, a suction ring with vacuum was aligned with corneal marks, and a safe distance for the docking interface was controlled with a joystick by the surgeon. After surgical site and depth was confirmed using the incorporated optical coherence tomography cross section, anterior capsulotomy and lens fragmentation were performed, followed by the ISAK. The programmed anterior capsulotomy size was 4.8 mm in diameter. Crystalline lens fragmentation was done using a standard template with a pattern described as “lens softening: quadrants” in the system.

ISAK nomogram of paired symmetric (same length) incisions centered on the steep corneal axis was done, with 8.0 mm in diameter and central 60% of total corneal thickness in depth remaining upper 20% and lower 20% without penetration. The arc length was determined by ISAK nomogram calculator v3, which has been provided on the website (http://www.femtoemulsification.com, v3.4, accessed November 1, 2018). The steep axis value to be entered in the calculator was decided from the automated keratometer. The length was proportional to preoperative corneal cylindrical power and was adjusted by the age and type of astigmatism, i.e., against-the-rule, with-the-rule, or oblique astigmatism. Foldable aspheric IOL (TECNIS 1-piece ZCB00 or PCB00 IOL; Abbot Medical Optics, Inc.) was implanted in patients of the AK group. The primary effectiveness target in femtosecond laser-assisted ISAK was refractive astigmatism for comparison with the toric IOL group. Corneal astigmatism was also evaluated.

In the toric IOL group, horizontal corneal-limbal marks using a sterile pen was made in each patient, while each was in a sitting position to avoid cyclotorsion. After a patient lied in a supine position in the operating room with aseptic drape, intraoperative markings were made under a surgical microscope with guidance from preoperative horizontal markings. After phacoemulsification, toric IOL (TECNIS Toric IOL; Abbott Medical Optics, Inc.) was implanted. The primary effectiveness target of toric IOL implantation was refractive astigmatism. The appropriate cylindrical power and axis placement of the toric IOL to be implanted were determined with the Barrett toric calculator (http://ascrs.org/tools/barrett-toric-calculator, accessed November 1, 2018). The IOL alignment and steep meridian value to be entered in the calculator were determined from the automated keratometer. After the implantation of IOL, IOL alignment and axis were re-checked.

The preoperative and postoperative (1-day, 1-month, 3-month, and 6-month) ophthalmic examinations included uncorrected distance visual acuity (UDVA) and corrected distance visual acuity (CDVA), autorefraction, manifest refraction, intraocular pressure measurements (non-contact tonometer; NT-530, NCT Nidek Co. Ltd., Aichi, Japan), dilated fundus examinations, and slit-lamp examinations (Haag-Streit, Gartenstadtstrasse, Köniz, Switzerland). Corneal astigmatism was measured by corneal topographies with the Orbscan (Bausch & Lomb, Rochester, NY, USA) and automated keratometer (KR-8800, Topcon Europe Medical BV). Additionally, for measuring ocular biometry such as axial length and anterior chamber depth, IOL Master 500 (Carl Zeiss Meditec, Oberkochen, Germany) was used.

Vector analysis of refractive astigmatism is based on the definitions and formulas given by Eydelman MB [23, 24]. We analyzed the intended refractive correction, surgically induced refractive correction, error ratio, correction ratio, error of magnitude, and error of angle. The intended refractive correction is the vector difference between the preoperative and the target postoperative cylinder vector. Assuming that target refractive state is emmetropia, it is equal to the preoperative astigmatism. The surgically induced refractive correction is defined as an amount and axis of astigmatism change achieved by surgery. The error ratio is the proportion of the intended correction that was not successfully corrected. The correction ratio is the ratio of the achieved correction magnitude to the targeted correction magnitude; whereas the error of magnitude is the difference in magnitudes between the surgically induced refractive correction and intended refractive correction. Finally, the error of angle is the angular difference between the achieved correction and the intended correction. Double-angle plots of preoperative corneal astigmatism and postoperative refractive astigmatism (3-month and 6-month) are drawn. The double-angle plot tool used in this paper is available on the American Society of Cataract and Refractive Surgery website (http://ascrs.org/tools/astigmatism-double-angle-plot-tool, accessed November 1, 2018) [25].

The Mann–Whitney *U* test, Wilcoxon signed-rank test, and Chi-square test were used for statistical analyses using the SPSS software version 21.0 (SPSS, Inc., Chicago, IL, USA). Differences were considered to be statistically significant for *P* values less than 0.05. Data from automated keratometer and topography were analyzed using vector analysis.

## Results

A total of 48 patients were identified in the study period. Of the 48 eyes of 48 patients, 27 eyes of 27 patients had undergone FLACS with ISAK, and 21 eyes of 21 patients had undergone conventional cataract surgery with toric IOL implantation. Demographics of the AK and toric IOL groups are summarized in Table 1. There were no significant differences in the mean age, preoperative UDVA, preoperative corneal astigmatism, and refractive astigmatism between the two groups. Preoperative CDVA was different between the two groups. No ocular complications, such as corneal ectasia or epithelial ingrowth, were reported in all cases.

**Table 1:** Demographics and clinical characteristics of the participants

	AK group	toric IOL group	*P*^*a*^
Number of eyes (n)	27	21	
Male/Female (% women)	9/18 (66.7)	8/13 (61.9)	0.732
Right/Left	18/9	10/11	0.184
Age (years)	69.4 ± 12.1 (39 to 89)	67.4 ± 12.5 (41 to 86)	0.581
ATR/WTR/Oblique	15/8/4	12/6/3	0.994
Preoperative UDVA (logMAR)	0.67 ± 0.442 (0.1 to 2.0)	0.43 ± 0.29 (0.15 to 1.3)	0.077
Preoperative CDVA (logMAR)	0.43 ± 0.39 (0.1 to 2.0)	0.22 ± 0.13 (0.1 to 0.6)	0.038
Corneal astigmatism from topography (D)	1.49 ± 0.36 (1.3 to 2.0)	1.60 ± 0.47 (1.2 to 2.0)	0.235
Corneal astigmatism from automated keratometer (D)	1.44 ± 0.39 (1.0 to 2.0)	1.50 ± 0.37 (0.87 to 2.0)	0.505
Refractive astigmatism (D)	1.85 ± 1.07 (0.87 to 3.50)	1.84 ± 0.81 (0.75 to 3.50)	0.723

Values are presented as mean ± standard deviation (range) or number

D = diopters; AK = arcuate keratotomy; IOL = intraocular lens; ATR = against-the-rule; WTR = with-the-rule; UDVA = uncorrected distance visual acuity; CDVA = corrected distance visual acuity; logMAR = logarithm of the minimum angle of resolution

^a^Differences of categorical variables assessed by Pearson’s chi-square test; continuous variables assessed by Mann-Whitney *U* test

**Fig. 1:**
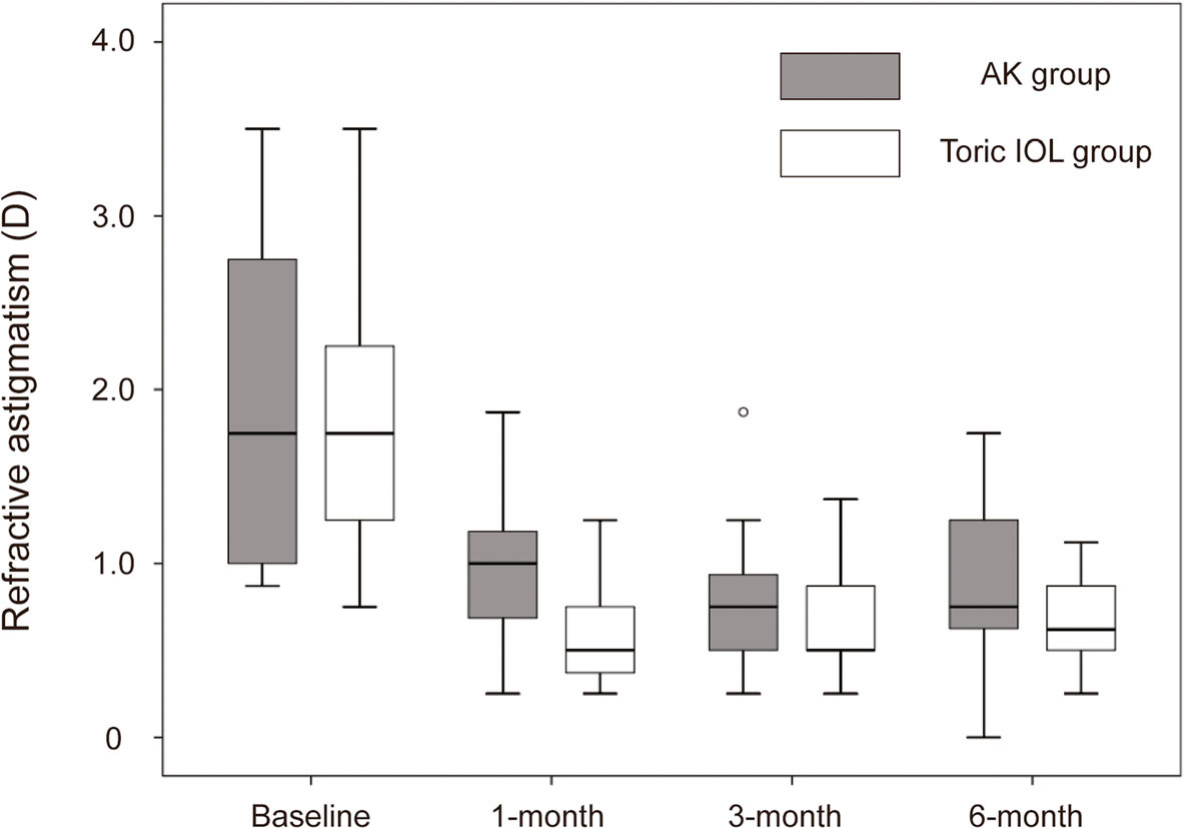
Box-whisker plots of refractive astigmatism by group and time

Table 2 demonstrates the results of refractive astigmatism and corneal astigmatism for both AK and toric IOL group. Significant reduction of refractive astigmatism was observed in both groups at 1-month, 3-month, and 6-month after the surgery, when compared with preoperative refractive astigmatism (1.85 ± 1.07 preoperatively, 0.96 ± 0.37 at 1-month, 0.76 ± 0.42 at 3-month, 0.99 ± 0.51 at 6-month postoperatively, ***P*** = 0.001, ***P*** < 0.001, ***P*** = 0.028, respectively, in AK group, and 1.84 ± 0.81 preoperatively, 0.63 ± 0.32 at 1-month, 0.67 ± 0.34 at 3-month, 0.68 ± 0.21 at 6-month postoperatively, ***P*** = 0.001, ***P*** = 0.001, ***P*** = 0.001, respectively, in toric IOL group; Table 2). There was no significant difference of refractive astigmatism between the two groups at 3-month and 6-month after surgery (0.76 ± 0.42 in AK group vs 0.67 ± 0.34 in toric IOL group, ***P*** = 0.483, at 3-month, and 0.99 ± 0.51 in AK group vs 0.68 ± 0.21 in toric IOL group, ***P*** = 0.057, at 6-month postoperatively; Table 2). Figure 1 shows a box and whisker plot of refractive astigmatism by group and time. The median refractive astigmatism had been significantly corrected postoperatively compared to baseline in both groups. The AK group showed larger interquartile ranges 1- and 6-month postoperatively compared to the toric IOL group. The corneal astigmatism was significantly reduced only in the AK group at 1-month, 3-month, and 6-month after the surgery, when compared with preoperative corneal astigmatism (automated keratometer: *P* = 0.001, 0.009, and 0.013 at 1-, 3-, and 6-month postoperatively, respectively; topography: *P* < 0.001, *P* = 0.003, *P* = 0.010 at 1-, 3-, and 6-month postoperatively, respectively; Table 2). Between two groups, significant difference of corneal astigmatism at all of each postoperative follow-up period was observed (Table 2).

**Table 2:** Results of corneal astigmatism and refractive astigmatism

	AK group	*P*^*a*^	toric IOL group	*P*^*a*^	*P*^*b*^
Manifest refractive astigmatism (D)					
Baseline	1.85 ± 1.07		1.84 ± 0.81		0.723
1-month postop	0.96 ± 0.37		0.62 ± 0.32		0.024
3-month postop	0.76 ± 0.42		0.67 ± 0.34		0.483
6-month postop	0.99 ± 0.51		0.68 ± 0.21		0.057
1-month vs baseline		0.001		0.001	
3-month vs baseline		0.000		0.001	
6-month vs baseline		0.028		0.001	
Corneal astigmatism from automated keratometer (D)					
Baseline	1.44 ± 0.39		1.50 ± 0.37		0.505
1-month postop	0.91 ± 0.37		1.22 ± 0.47		0.045
3-month postop	0.99 ± 0.67		1.35 ± 0.33		0.021
6-month postop	0.98 ± 0.69		1.37 ± 0.41		0.032
1-month vs baseline		0.001		0.114	
3-month vs baseline		0.009		0.306	
6-month vs baseline		0.013		> 0.999	
Corneal astigmatism from topography (D)					
Baseline	1.49 ± 0.36		1.60 ± 0.47		0.235
1-month postop	0.93 ± 0.44		1.40 ± 0.50		0.011
3-month postop	1.02 ± 0.52		1.50 ± 0.47		0.010
6-month postop	0.94 ± 0.40		1.53 ± 0.46		0.018
1-month vs baseline		< 0.001		0.048	
3-month vs baseline		0.003		0.058	
6-month vs baseline		0.010		0.149	

Values are presented as mean ± standard deviation

D = diopters; AK = arcuate keratotomy; IOL = intraocular lens

^a^Comparison with baseline within groups assessed by Wilcoxon signed-rank test

^b^Comparison between groups assessed by Mann-Whitney *U* test

Table 3 demonstrates the results of vector analysis of refractive astigmatism. There was no significant difference in all vector analysis parameters (surgically induced refractive correction, error ratio, correction ratio, error of magnitude, and error of angle) between the two groups at 1-month, 3-month, and 6-month after surgery (all ***P*** values > 0.05; Table 3) except error ratio at 1-months postoperatively (0.66 ± 0.44 in AK group vs 0.31 ± 0.21 in toric IOL group, ***P*** = 0.011; Table 3). In the AK group, there was no significant difference between the 1-month, 3-month, and 6-month postoperative vector analysis parameters (all ***P*** values > 0.05; Table 3). In the toric IOL group, there were significant differences between 1-month and 3-month and between 1-month and 6-month postoperatively in surgically induced refractive correction, correction ratio, and error of magnitude (1-month vs. 3-month and 1-month vs. 6-month, *P* = 0.006 and 0.009, respectively, in surgically induced refractive correction; 1-month vs. 3-month and 1-month vs. 6-month, *P* = 0.004 and 0.013, respectively, in the correction ratio; and 1-month vs. 3-month and 1-month vs. 6-month, *P* = 0.006 and 0.009, respectively, in the error of magnitude; Table 3).

**Table 3:** Vector analysis of refractive astigmatism

	AK group	*P*^*a*^	toric IOL group	*P*^*a*^	*P*^*b*^
Surgically induced refractive correction (D)					
1-month postop	1.49 ± 1.12		1.69 ± 0.84		0.211
3-month postop	1.57 ± 1.51		1.49 ± 0.75		0.465
6-month postop	1.36 ± 0.93		1.40 ± 0.87		0.973
1-month vs 3-month		0.884		0.006	
3-month vs 6-month		0.794		0.807	
1-month vs 6-month		0.401		0.009	
Error ratio					
1-month postop	0.66 ± 0.44		0.31 ± 0.21		0.011
3-month postop	0.60 ± 0.44		0.37 ± 0.10		0.071
6-month postop	0.69 ± 0.58		0.39 ± 0.30		0.105
1-month vs 3-month		0.469		0.552	
3-month vs 6-month		0.222		0.965	
1-month vs 6-month		0.463		0.414	
Correction ratio					
1-month postop	0.80 ± 0.40		0.99 ± 0.25		0.108
3-month postop	0.82 ± 0.54		0.81 ± 0.23		0.465
6-month postop	0.78 ± 0.43		0.78 ± 0.32		0.840
1-month vs 3-month		0.931		0.004	
3-month vs 6-month		0.809		0.650	
1-month vs 6-month		0.681		0.013	
Error of magnitude (D)					
1-month postop	0.31 ± 0.62		0.05 ± 0.55		0.194
3-month postop	0.24 ± 1.18		0.34 ± 0.51		0.808
6-month postop	0.47 ± 0.70		0.44 ± 0.69		0.813
1-month vs 3-month		0.884		0.006	
3-month vs 6-month		0.794		0.807	
1-month vs 6-month		0.401		0.009	
Error of angle (°)					
1-month postop	15.13 ± 67.82		7.01 ± 75.03		0.820
3-month postop	−0.03 ± 75.59		−4.42 ± 82.27		0.697
6-month postop	6.00 ± 65.83		−12.96 ± 74.47		0.522
1-month vs 3-month		0.426		0.594	
3-month vs 6-month		0.968		0.650	
1-month vs 6-month		0.575		0.221	

Values are presented as mean ± standard deviation

D = diopters; AK = arcuate keratotomy; IOL = intraocular lens

^a^Comparison between follow-up period within groups assessed by Wilcoxon signed-rank test

^b^Comparison between groups assessed by Mann-Whitney *U* test

**Fig. 2:**
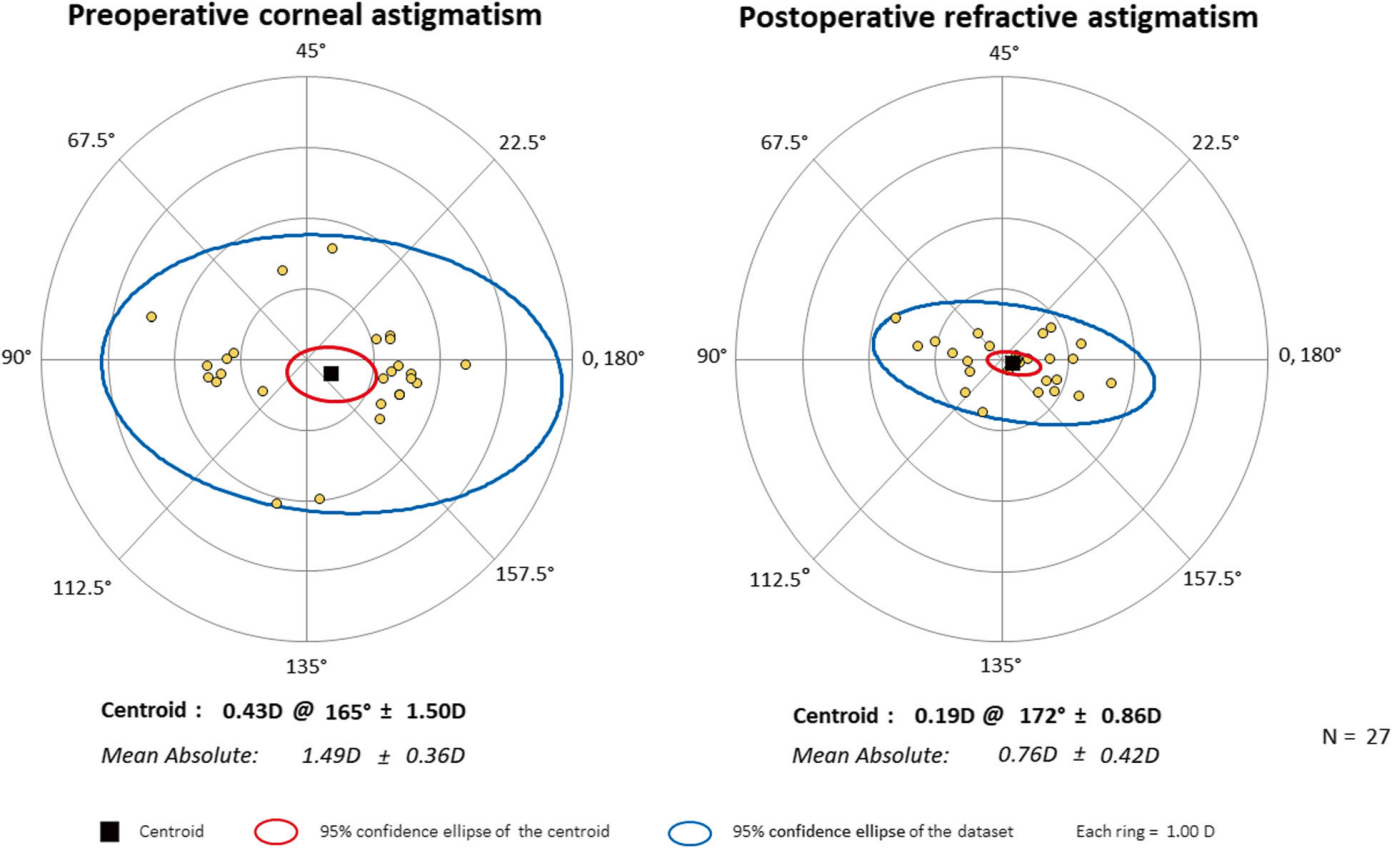
Double-angle plots of preoperative corneal astigmatism and 3-month postoperative refractive astigmatism in patients who underwent femtosecond laser-assisted cataract surgery with intrastromal arcuate keratotomy

**Fig. 3:**
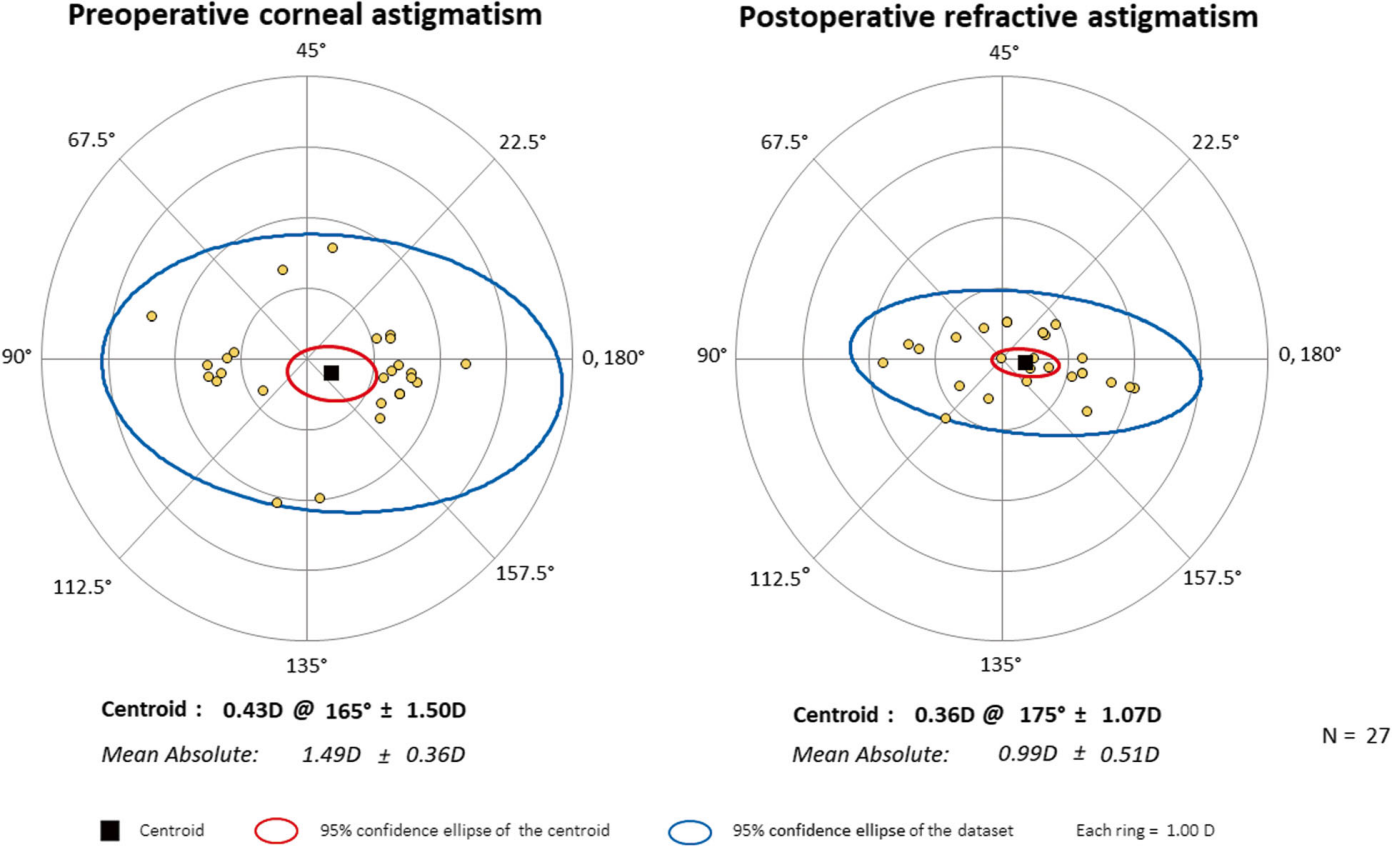
Double-angle plots of preoperative corneal astigmatism and 6-month postoperative refractive astigmatism in patients who underwent femtosecond laser-assisted cataract surgery with intrastromal arcuate keratotomy

**Fig. 4:**
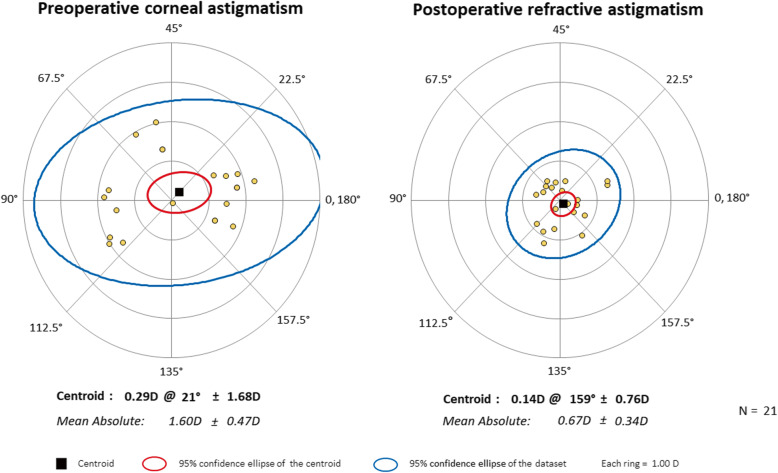
Double-angle plots of preoperative corneal astigmatism and 3-month postoperative refractive astigmatism in patients who underwent cataract surgery with toric intraocular lens implantation

**Fig. 5:**
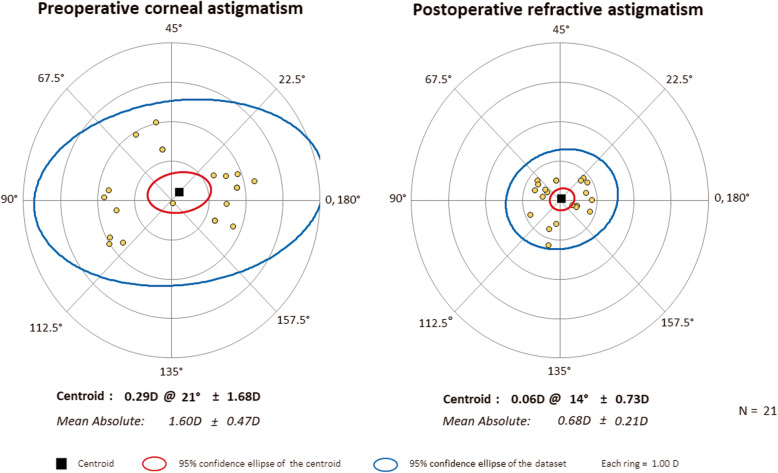
Double-angle plots of preoperative corneal astigmatism and 6-month postoperative refractive astigmatism in patients who underwent cataract surgery with toric intraocular lens implantation

Figures show double-angle plots of preoperative corneal astigmatism and postoperative refractive astigmatism at 3-month and 6-month after surgery in both AK group (Figs. 2 and 3) and toric IOL group (Figs. 4 and 5). Effective postoperative astigmatism reduction was observed in all of the double-angle plots.

There was no intraoperative complication requiring secondary operation in both groups. In the AK group, no case of corneal ectasia or corneal perforation was occurred. There was no instance of IOL misalignment more than 10° in the toric IOL group.

## Discussion

Cataract surgery has evolved into a refractive procedure during which spherical and cylindrical errors are minimized, finally resulting in satisfactory spectacle-free visual outcomes. However, accurate surgical planning and perfect correction of astigmatism have been challenging for cataract surgeons. Many previous studies compared several options during cataract surgery to correct astigmatism, including opposite clear corneal incisions, limbal relaxing incisions, manual AK, and toric IOL implantation. The implantation of toric IOL is considered as an effective surgical option to reduce refractive astigmatism [14, 26, 27]. Additionally, FLACS showed comparable results with conventional cataract surgery [28]. Furthermore, simultaneous astigmatism correction with FLACS with ISAK is reported to be adjustable for managing astigmatism in cataract surgery [29]. We previously reported a comparison between femtosecond laser-assisted trans-epithelial AK after cataract surgery for residual astigmatism and cataract surgery with toric IOL implantation [19]. To the best of our knowledge, no comparison between FLACS with ISAK and toric IOL implantation in the aspect of astigmatism correction was reported yet. In the present study, we aim to compare the effectiveness of astigmatism reduction in cataract surgery between the two procedures: simultaneous FLACS with ISAK versus toric IOL implantation with conventional cataract surgery.

In this study, significant correction of refractive astigmatism was derived by both FLACS with ISAK and toric IOL implantation. Our previous study suggested that the femtosecond laser-assisted trans-epithelial AK following conventional cataract surgery is comparable to implantation of toric IOL in patients with preoperative astigmatism between + 1.00 and + 3.00 D [19]. However, there are some important differences between the current study and our previous study. That is, whereas femtosecond laser-assisted trans-epithelial AK was performed for patients who were not satisfied with their residual astigmatism, following conventional cataract surgery in our previous study, [19] femtosecond laser-assisted ISAK was performed simultaneously with FLACS in patients with lower preexisting astigmatism up to + 2.00 D in the present study. In addition, ISAK was performed in this study, while trans-epithelial AK was done previously. ISAK with central 60% of total corneal thickness in depth remaining upper 20% and lower 20% without penetration could be safer than trans-epithelial AK which includes Bowman’s layer incision followed by anterior stromal inflammation with epithelial hyperplasia [30]. According to our previous study, in cases of lower magnitude of preoperative astigmatism, there was comparable effect between femtosecond laser-assisted trans-epithelial AK and toric IOL implantation with regards to postoperative astigmatism correction. In the current study, also, comparable astigmatism correction was noted between simultaneous FLACS with ISAK versus toric IOL implantation with conventional phacoemulsification in terms of refractive astigmatism reduction after cataract surgery.

By performing femtosecond laser-assisted ISAK during cataract surgery, corneal astigmatism was effectively corrected postoperatively. Significant reduction in corneal astigmatism was made in the AK group at all of each postoperative follow-up period. Principle of the toric IOL implantation does not affect corneal astigmatism. However, for the reason that on-axis clear corneal incision may alter postoperative surgically induced astigmatism in cataract surgery, corneal astigmatism as well as refractive astigmatism was also compared between the two groups. In the toric IOL group, the mean reduction of corneal astigmatism was 0.20 D from topography and 0.32 D from the automated keratometer at the 1-month follow-up, which dropped to 0.07 D from topography and 0.13 D from the automated keratometer at the 6-month follow-up. These changes can be attributed to the effect of surgically induced astigmatism, and our results are consistent with results of previous studies [31, 32]. Obviously, compared to baseline preoperative levels, the amount of reduction was not significant in the toric IOL group, while significant reduction of corneal astigmatism was noted in the AK group.

The vector analysis showed better stability of astigmatism reduction in the toric IOL group compared to the AK group. In the AK group, no significant change was seen in vector analysis during each follow-up period of 1-month, 3-month, and 6-month postoperatively***.*** In line with our results, previous study reported that the reduced corneal astigmatism after ISAK showed a stable course during 6-month postoperatively [16]. Reported stability in astigmatism correction could be attributed to the fact that femtosecond laser-assisted ISAK is performed with optical coherence tomography-guided computer software with calculated nomogram. Considering that there was no ocular complication such as corneal ectasia or epithelial ingrowth, albeit short term follow-up period, FLACS with ISAK can be an effective, reproducible, and precise alternative procedure not only to remove cataract, but also decrease astigmatism. Compared to the AK group, the error ratio and amount of postoperative refractive astigmatism at all follow-ups seemed to be better with smaller variability in the toric IOL group. These findings suggest better stability of astigmatic correction in toric IOL implantation with conventional cataract surgery compared to FLACS with ISAK.

Between the two groups, no significant difference was observed in the correction ratio postoperatively. These findings with respect to undercorrection suggest that the AK group provides comparable outcomes in astigmatic correction compared to the toric IOL group. The mean correction ratio of ISAK during FLACS in the current study was 0.82 after 3 months and 0.78 after 6 months. Byun et al. [33] previously reported a similar value of 0.72 after 3 months and 0.87 after 6 months, and Nick et al. [34] reported a mean correction ratio of 0.80 after 1 year. The current and previous studies used the same nomogram [21] of ISAK during FLACS, and these outcomes indicating undercorrection connote the need to adjust the nomogram. Rotation of IOL could also have contributed to the tendency of undercorrection in the toric IOL group. Previous studies suggest that the rotation of toric IOL contributes to the reduced correction of astigmatism [26, 35]. Considering that 1° of off-axis IOL rotation is estimated to cause a reduction of up to 3.3% of IOL cylindrical power, rotation of toric IOL is very crucial to maintain UDVA and visual quality. In our study, about 0.2 D of cylindrical power correction was reduced from 1-month to 3-month postoperatively in the toric IOL group, which corresponds to approximately 10% of preoperative cylindrical power. Roughly 3° rotation can be presumed, which corresponds to similar outcomes with the study by Miyake T et al. [26], which suggests a 2.2° rotation 1-week to 3 month postoperatively. Rotational stability of toric IOL should have been accounted for; however, the IOL alignment or axis was not assessed at each follow-up, which is a limitation of this study. Clinically, this minimal rotation may not significantly affect UDVA or patients’ satisfaction. Nevertheless, careful routine check-up is needed in patients with toric IOL implantation.

The present study had several limitations, including its retrospective design and relatively short-term follow-up period up to only 6 months postoperatively. Long-term results would allow a more thorough examination of corneal astigmatism changes, which could be derived from the healing response after ISAK. This study included a small sample size, making it challenging to represent definite conclusions. Despite the limited statistical power, this study represents comparable outcomes of ISAK in FLACS in terms of astigmatism correction compared to conventional cataract surgery with toric IOL implantation. A prospective randomized clinical trial study with an appropriate sample size would allow a more thorough comparison with regards to astigmatism correction between the FLACS with ISAK and implantation of toric IOL during cataract surgery.

## Conclusions

In conclusion, when compared with the toric IOL implantation, the ISAK in FLACS could be a comparable procedure in terms of reducing astigmatism in cataract surgery for patients with moderate astigmatism, although toric IOL implantation seems to be more stable.

## Data Availability

Data are available upon reasonable request from the authors.

## Abbreviations

FLACS: Femtosecond laser-assisted cataract surgery
ISAK: Intrastromal arcuate keratotomy
AK: arcuate keratotomy
IOL: Intraocular lens
UDVA: Uncorrected distance visual acuity
CDVA: Corrected distance visual acuity
